# The vitamin C paradigm: new frontiers in blood transfusion

**DOI:** 10.1097/MS9.0000000000003018

**Published:** 2025-05-21

**Authors:** Emmanuel Ifeanyi Obeagu

**Affiliations:** Department of Biomedical and Laboratory Science, Africa University, Mutare, Zimbabwe

**Keywords:** antioxidant therapy, blood storage, erythrocyte preservation, transfusion medicine, vitamin C

## Abstract

Vitamin C, a potent antioxidant, is gaining attention in the field of transfusion medicine for its potential to enhance blood transfusion practices. Recent studies suggest that vitamin C can significantly improve erythrocyte preservation by mitigating oxidative damage during storage, thereby maintaining cell membrane integrity and functionality. This preservation is crucial for ensuring the efficacy and safety of transfused blood, ultimately leading to better patient outcomes. In addition to its role in erythrocyte preservation, vitamin C has been shown to modulate immune responses, which can be particularly beneficial in reducing the risks associated with transfusion-related immunomodulation (TRIM). By influencing both innate and adaptive immunity, vitamin C can help mitigate adverse immune reactions and improve the overall clinical outcomes for transfusion recipients. These immune-modulating properties underscore the potential of vitamin C to address some of the critical challenges in transfusion medicine. Furthermore, clinical trials have demonstrated that the incorporation of vitamin C in transfusion protocols can lead to enhanced recovery rates and reduced incidence of transfusion-related complications. The multifaceted benefits of vitamin C, including its antioxidant defense, immune support, and metabolic regulation, highlight its promise as a transformative agent in blood transfusion practices. As research continues to uncover the optimal use of vitamin C in this context, it is poised to become a pivotal element in improving transfusion efficacy and patient care.

## Introduction

Transfusion medicine, a cornerstone of modern healthcare, plays an essential role in managing various medical conditions ranging from acute blood loss to chronic anemia and hematologic disorders. The success of blood transfusions hinges on the quality and efficacy of the blood products administered. In this regard, the preservation of erythrocytes (red blood cells) during storage and their functionality post-transfusion are critical factors that influence patient outcomes. Recent advancements in the field have highlighted the potential of vitamin C as a significant contributor to improving these aspects of transfusion medicine^[1,2].^ Vitamin C, also known as ascorbic acid, is a water-soluble vitamin renowned for its potent antioxidant properties^[3]^. It is essential for various physiological functions, including collagen synthesis, wound healing, and immune function. Its role in protecting cells from oxidative damage has been extensively studied, particularly in the context of red blood cells, which are highly susceptible to oxidative stress due to their high iron content and constant exposure to oxygen.Oxidative stress is a major concern in the storage of blood products^[4]^. During storage, erythrocytes undergo biochemical and structural changes, collectively referred to as storage lesions. These changes include lipid peroxidation, protein oxidation, and a decline in ATP levels, leading to decreased cell viability and functionality. The infusion of oxidatively damaged erythrocytes can result in poor transfusion outcomes, including reduced cell survival in circulation and an increased risk of adverse reactions. Incorporating antioxidants like vitamin C into storage solutions can potentially mitigate these storage lesions, preserving erythrocyte quality. The immunomodulatory effects of vitamin C further extend its relevance to transfusion medicine^[5]^. TRIM is a phenomenon where the recipient’s immune system is altered following the administration of allogeneic blood products. This can lead to a range of clinical complications, including increased susceptibility to infections, alloimmunization, and even tumor progression in cancer patients. By modulating the immune response, vitamin C can help reduce the incidence and severity of TRIM, thereby enhancing the safety of blood transfusions.

**Highlights**

- Research unveils vitamin C’s transformative potential in blood transfusion, enhancing blood storage and patient outcomes.
- Innovations in vitamin C applications promise to extend blood shelf-life, reducing waste and shortages.
- Breakthroughs reveal vitamin C’s role in preserving red blood cells, ensuring safer transfusions.
- Clinical trials demonstrate improved recovery rates and reduced complications with vitamin C-enriched transfusions.
- The paradigm shift positions vitamin C at the forefront of modern transfusion medicine, heralding a new era of healthcare.

Clinical evidence supporting the benefits of vitamin C in transfusion medicine is growing. Several studies have reported improved patient outcomes associated with vitamin C-enriched transfusions^[6,7]^.These benefits include enhanced recovery rates, reduced incidence of transfusion-related complications, and better overall prognoses. The mechanisms underlying these improvements are multifaceted, involving antioxidant protection, immune support, and metabolic regulation.The potential of vitamin C to transform transfusion practices extends beyond erythrocyte preservation and immune modulation^[8]^. It also encompasses the broader scope of patient care, including reducing the overall burden of transfusion-related complications and improving the efficiency and effectiveness of blood supply management. This holistic approach can lead to significant advancements in the field, ultimately benefiting a wide range of patients who rely on transfusions for their health and well-being.Innovations in transfusion medicine, such as the inclusion of vitamin C, also have implications for the healthcare system at large. By improving transfusion outcomes and reducing complications, healthcare providers can achieve better resource utilization, lower healthcare costs, and enhance patient satisfaction. This aligns with the broader goals of healthcare systems to provide high-quality, cost-effective care while improving patient outcomes^[8,9]^.

## Aim

The aim of this study is to explore and evaluate the potential of vitamin C as a therapeutic adjunct in transfusion medicine, focusing on its benefits, challenges, and future research opportunities.

## Historical perspective of vitamin C in blood transfusion

The discovery and understanding of vitamin C (ascorbic acid) can be traced back to the early 20th century when it was identified as the essential nutrient preventing scurvy^[9]^. The connection between vitamin C and blood health has long been recognized, given its crucial role in collagen synthesis, immune function, and as an antioxidant. However, the specific application of vitamin C in transfusion medicine is a relatively recent development that has evolved over the past few decades.In the early years of transfusion medicine, the primary focus was on the safety and compatibility of blood products, with less emphasis on the biochemical changes that occur during storage^[10]^. The phenomenon of storage lesions – biochemical and structural changes in stored erythrocytes – was well-documented, but the role of antioxidants in mitigating these changes was not fully explored. Early studies in the mid-20th century began to highlight the importance of oxidative stress in the degradation of stored blood products, setting the stage for future research into potential solutions. The interest in using antioxidants to preserve blood products gained momentum in the late 20th and early 21st centuries^[11]^. Initial research focused on various antioxidants, including vitamin E and glutathione. However, it was vitamin C’s potent antioxidant properties and its essential role in maintaining cellular health that drew significant attention. Early experimental studies demonstrated that vitamin C could reduce oxidative damage in erythrocytes, suggesting its potential for improving the quality of stored blood.

As researchers delved deeper into the biochemistry of blood storage, it became evident that oxidative damage was a major contributor to the decline in erythrocyte quality over time. Studies in the late 1990s and early 2000s began to explore the specific effects of vitamin C on erythrocyte preservation^[12]^. These studies showed that vitamin C could effectively reduce hemolysis, maintain cell membrane integrity, and improve the overall viability of stored erythrocytes. Such findings were crucial in demonstrating the practical benefits of vitamin C in transfusion medicine. The potential of vitamin C in transfusion medicine was further underscored by research into its broader physiological effects^[13]^. Beyond its role as an antioxidant, vitamin C was found to play a vital role in modulating immune responses. This discovery was significant given the challenges associated with transfusion-related immunomodulation (TRIM), where the recipient’s immune system is altered following the transfusion of allogeneic blood products. By supporting immune function and reducing oxidative stress, vitamin C emerged as a multifaceted agent capable of addressing several key issues in transfusion medicine.

The integration of vitamin C into clinical practice, however, has been gradual. Initial clinical trials focused on the safety and efficacy of vitamin C-enriched transfusions. These studies aimed to determine optimal dosing, administration methods, and potential side effects. Results from these trials have generally been positive, showing improved patient outcomes, reduced incidence of transfusion-related complications, and better overall prognoses. Despite these promising results, the widespread adoption of vitamin C in transfusion protocols has been slow, primarily due to the need for more extensive research and standardized guidelines. In recent years, the field has seen a resurgence of interest in the potential benefits of vitamin C in transfusion medicine^[14]^. Advances in molecular biology and immunology have provided deeper insights into the mechanisms through which vitamin C exerts its effects. Modern research continues to build on the foundational studies of the late 20th century, exploring new applications and optimizing protocols to maximize the benefits of vitamin C in transfusion settings. The historical evolution of vitamin C in transfusion medicine reflects a broader trend in medical science: the shift from purely symptomatic treatment to a more holistic approach that considers the underlying biochemical and physiological processes. This paradigm shift is evident in the growing body of research that supports the use of vitamin C not only as a preservative for stored blood but also as a therapeutic agent that can enhance patient outcomes in various clinical scenarios.

## The potential benefits of vitamin C in transfusion medicine

Vitamin C, a powerful antioxidant, holds significant potential in transforming transfusion medicine. Its diverse roles in enhancing erythrocyte preservation, modulating immune responses, and improving clinical outcomes make it a promising agent in addressing key challenges in this field.

## Enhanced erythrocyte preservation

One of the primary benefits of vitamin C in transfusion medicine is its ability to enhance erythrocyte preservation^[15]^. During storage, red blood cells (RBCs) undergo oxidative stress, leading to lipid peroxidation, protein oxidation, and membrane damage. These storage lesions compromise the viability and functionality of RBCs, reducing their efficacy post-transfusion. Vitamin C mitigates oxidative damage by neutralizing reactive oxygen species (ROS), thus preserving cell membrane integrity and reducing hemolysis. Studies have demonstrated that RBCs stored with vitamin C exhibit lower levels of oxidative damage, better ATP maintenance, and prolonged shelf life, ensuring higher quality and efficacy of transfused blood.

## Improved immune modulation

TRIM is a phenomenon where the recipient’s immune system is altered post-transfusion, leading to potential complications such as increased susceptibility to infections and alloimmunization^[16]^. Vitamin C’s immune-modulating properties can help address these issues. It supports the function of various immune cells, including neutrophils, lymphocytes, and macrophages, enhancing their ability to respond to infections and inflammation. By modulating cytokine production and reducing oxidative stress, vitamin C can minimize the adverse immune responses associated with blood transfusions, thereby improving the overall safety and efficacy of the procedure.

## Reduction of transfusion-related complications

Transfusions can sometimes lead to complications such as transfusion-related acute lung injury (TRALI), transfusion-associated circulatory overload (TACO), and febrile non-hemolytic transfusion reactions (FNHTRs)^[17]^. The anti-inflammatory and antioxidant effects of vitamin C can help reduce the incidence and severity of these complications. For instance, by decreasing the production of pro-inflammatory cytokines and ROS, vitamin C can mitigate the inflammatory responses that contribute to TRALI and FNHTRs. This protective effect enhances patient safety and outcomes following transfusions.

## Enhanced clinical outcomes

Clinical studies have shown that patients receiving vitamin C-enriched transfusions exhibit improved recovery rates and overall prognoses. The antioxidant and immune-modulating properties of vitamin C contribute to faster recovery and reduced incidence of infections and other complications. For example, in critically ill patients or those undergoing major surgeries, vitamin C supplementation has been associated with decreased length of hospital stay and lower mortality rates. These findings suggest that incorporating vitamin C into transfusion protocols can lead to better clinical outcomes and improved patient care^[16]^.

## Support for patients with chronic conditions

Patients with chronic conditions such as anemia, thalassemia, and sickle cell disease often require multiple transfusions. The repeated administration of oxidatively damaged RBCs can exacerbate their condition and lead to iron overload and other complications. Vitamin C’s role in preserving RBC quality and reducing oxidative stress can provide significant benefits for these patients. By ensuring the transfusion of high-quality blood, vitamin C can help manage chronic conditions more effectively, improving patients’ quality of life and reducing the burden of transfusion-related complications^[16]^.

## Cost-effectiveness and resource utilization

Improving the quality of stored blood with vitamin C can also have economic benefits. Enhanced RBC preservation reduces the wastage of blood products, leading to more efficient use of donated blood. This is particularly important in healthcare systems facing shortages of blood supply. Additionally, by reducing transfusion-related complications and improving patient outcomes, vitamin C supplementation can lower healthcare costs associated with extended hospital stays and additional treatments. This cost-effectiveness makes vitamin C an attractive option for healthcare providers and policymakers^[15]^.

## Broader implications for blood transfusion practices

The integration of vitamin C into transfusion medicine aligns with the broader goals of improving patient care and outcomes. It represents a shift toward a more holistic approach that considers the biochemical and physiological aspects of blood storage and transfusion. By addressing oxidative stress and immune modulation, vitamin C supplementation can lead to significant advancements in transfusion practices, ultimately benefiting a wide range of patients who rely on transfusions for their health and well-being^[15]^.

## Potential synergistic effects with other therapies

Vitamin C’s multifaceted benefits suggest potential synergistic effects when used in combination with other therapies. For instance, combining vitamin C with other antioxidants or anti-inflammatory agents could enhance the overall protective effects against oxidative stress and inflammation. Such combination therapies could further improve the quality of stored blood and the outcomes of transfusions, providing a comprehensive strategy for addressing the challenges in transfusion medicine^[16]^.

## Antioxidant properties and erythrocyte preservation

Erythrocytes, or red blood cells, are crucial for oxygen transport in the body, and their integrity and functionality are paramount in transfusion medicine. During storage, erythrocytes undergo oxidative stress, leading to cellular damage that compromises their efficacy post-transfusion. Vitamin C, known for its potent antioxidant properties, offers a promising solution to mitigate oxidative damage and enhance erythrocyte preservation. Oxidative stress during erythrocyte storage arises from the accumulation of reactive ROS, which cause lipid peroxidation, protein oxidation, and DNA damage^[18]^. These changes, collectively termed storage lesions, result in reduced cell membrane integrity, hemolysis, and decreased cell viability. As a consequence, transfused erythrocytes may exhibit shortened lifespan in circulation, diminished oxygen-carrying capacity, and increased risk of adverse reactions in recipients. Addressing oxidative stress is thus critical for improving the quality of stored blood products.

Vitamin C’s primary role as an antioxidant involves neutralizing ROS and preventing oxidative damage^[19]^. It acts as a reducing agent, donating electrons to free radicals and converting them into less reactive species. In erythrocytes, vitamin C regenerates other antioxidants, such as vitamin E, from their oxidized forms, thereby maintaining a robust cellular antioxidant defense system. This synergistic effect enhances the overall protective capacity against oxidative stress. Research has demonstrated that the inclusion of vitamin C in blood storage solutions can significantly reduce the extent of storage lesions^[20]^. Studies have shown that erythrocytes stored with vitamin C exhibit lower levels of lipid peroxidation and hemolysis compared to those stored without it^[21,22]^. Additionally, vitamin C helps maintain ATP levels and cell membrane integrity, further contributing to the preservation of erythrocyte functionality. The beneficial effects of vitamin C on erythrocyte preservation extend to the post-transfusion phase as well. Transfused erythrocytes that are less oxidatively damaged are more likely to survive longer in the recipient’s circulation, providing better therapeutic outcomes. This is particularly important in patients requiring multiple transfusions or those with conditions such as chronic anemia, where the efficiency of each transfusion is crucial.

Beyond its direct antioxidant effects, vitamin C influences other cellular processes that contribute to erythrocyte preservation^[23]^. It plays a role in collagen synthesis, which is essential for maintaining the structural integrity of blood vessels and supporting proper circulation. By promoting vascular health, vitamin C indirectly supports the optimal function and longevity of transfused erythrocytes. The application of vitamin C in erythrocyte preservation also aligns with broader efforts to improve the safety and efficacy of blood transfusions^[17]^. Traditional methods of blood storage and preservation have focused on temperature control and the use of additives to prolong shelf life. The addition of antioxidants like vitamin C represents a complementary approach that addresses the biochemical aspects of erythrocyte degradation, offering a more comprehensive strategy for enhancing blood quality. Despite the promising findings, the routine use of vitamin C in blood storage solutions is not yet standard practice. Further research is needed to establish optimal concentrations, dosing regimens, and administration protocols. Additionally, large-scale clinical trials are required to confirm the benefits observed in laboratory settings and to assess potential risks or side effects associated with vitamin C supplementation in transfusion medicine.

## Immune modulation

The immune system plays a critical role in determining the success and safety of blood transfusions. TRIM is a phenomenon where the recipient’s immune response is altered following the administration of allogeneic blood products, potentially leading to various complications such as increased susceptibility to infections, alloimmunization, and adverse transfusion reactions^[24]^. Vitamin C, with its well-documented immune-modulating properties, offers significant potential to mitigate these issues and enhance the overall safety and efficacy of blood transfusions. Vitamin C is essential for the optimal function of the innate immune system, the body’s first line of defense against pathogens^[25]^. It supports various cellular activities of the innate immune response, including the function of phagocytes, which are critical for engulfing and destroying pathogens. By maintaining the oxidative burst capability of neutrophils and enhancing their chemotaxis and phagocytosis, vitamin C helps ensure a robust initial immune response following transfusion. This can be particularly beneficial in preventing infections in immunocompromised patients or those undergoing surgeries where the risk of infection is high. The adaptive immune system, which provides long-term immunity and immunological memory, also benefits from vitamin C supplementation^[26]^. It enhances the proliferation and differentiation of B- and T-lymphocytes, key components of the adaptive immune response. By supporting the production and function of these lymphocytes, vitamin C can help mitigate the immune alterations associated with TRIM. This is crucial for preventing alloimmunization, where the recipient’s immune system mounts a response against donor antigens, potentially leading to complications in future transfusions.

Inflammation is a common response following blood transfusion and can lead to complications such as TRALI and FNHTRs^[27]^. Vitamin C’s anti-inflammatory properties can help reduce the production of pro-inflammatory cytokines and oxidative stress, both of which contribute to these adverse reactions. By modulating the inflammatory response, vitamin C not only enhances patient comfort but also reduces the risk of severe complications, thereby improving overall transfusion outcomes. Cytokines are signaling molecules that play a vital role in the immune response. Dysregulated cytokine production can lead to immune system imbalances and adverse reactions post-transfusion^[28]^. Vitamin C has been shown to influence cytokine production, promoting a balanced immune response. It enhances the production of anti-inflammatory cytokines while reducing pro-inflammatory cytokines, thus maintaining immune homeostasis. This modulation is particularly beneficial in preventing cytokine storms, which can be life-threatening in severe transfusion reactions.

Immune cells are highly active and thus produce a significant amount of ROS during their response to pathogens. While ROS play a role in pathogen destruction, excessive ROS can lead to oxidative damage to immune cells themselves, impairing their function^[29]^. Vitamin C, through its antioxidant properties, protects immune cells from oxidative damage, ensuring their optimal function. This protection is crucial for maintaining a robust immune response and preventing the immunosuppression that can occur following transfusions. Immunosuppression is a significant concern following blood transfusion, as it can increase the recipient’s vulnerability to infections and other complications. Vitamin C has been shown to prevent immunosuppression by supporting the function and proliferation of immune cells^[30]^. By maintaining immune competence, vitamin C helps ensure that the recipient’s immune system remains vigilant and responsive to potential threats, thus reducing the risk of post-transfusion infections and other related complications.

The immune-modulating effects of vitamin C can be enhanced when used in combination with other nutrients, such as vitamin E and zinc, which also play crucial roles in immune function^[31]^. For example, vitamin C can regenerate oxidized vitamin E, thereby enhancing its antioxidant capacity. Such synergistic effects can provide a more comprehensive approach to supporting the immune system and mitigating TRIM, ultimately improving the safety and efficacy of blood transfusions. Numerous clinical studies have demonstrated the immune-modulating benefits of vitamin C in various settings^[5,32]^. For instance, patients with sepsis or those undergoing major surgeries have shown improved immune responses and reduced complications with vitamin C supplementation. These findings suggest that similar benefits could be realized in transfusion medicine, where immune modulation plays a critical role in patient outcomes. Continued research and clinical trials are necessary to confirm these benefits and establish standardized protocols for vitamin C use in transfusions. The integration of vitamin C into transfusion protocols holds significant implications for transfusion medicine. By reducing the incidence and severity of TRIM and other transfusion-related complications, vitamin C can enhance patient safety and outcomes. This improved safety profile can also lead to increased confidence in blood transfusions among patients and healthcare providers, promoting better overall care.

## Clinical outcomes

In transfusion medicine, the ultimate measure of success lies in improved clinical outcomes for patients. Vitamin C, with its robust antioxidant and immune-modulating properties, has shown considerable promise in enhancing these outcomes^[33]^. The incorporation of vitamin C into transfusion protocols can lead to a range of clinical benefits, including reduced complications, improved recovery rates, and better overall patient prognoses. Transfusion-related complications, such as TRALI, TACO, and FNHTRs, pose significant risks to patients. Vitamin C’s antioxidant and anti-inflammatory properties can help mitigate these complications. By reducing oxidative stress and modulating inflammatory responses, vitamin C lowers the likelihood and severity of these adverse events. Clinical studies have shown that patients receiving vitamin C-enriched transfusions experience fewer incidences of these complications, contributing to safer transfusion practices and improved patient safety.^[34-36]^Patients undergoing major surgeries or those with conditions requiring frequent transfusions often face prolonged recovery periods. Vitamin C can expedite recovery by supporting immune function, reducing oxidative damage, and enhancing wound healing. For example, in surgical patients, vitamin C supplementation has been associated with reduced post-operative infections and quicker healing times. In critically ill patients, vitamin C has been shown to improve recovery rates by bolstering the immune system and reducing the overall inflammatory burden. These benefits translate to shorter hospital stays, decreased need for additional medical interventions, and faster return to normal activities^[37]^.

Critically ill patients, such as those in intensive care units (ICUs), are particularly vulnerable to complications from oxidative stress and immune dysregulation. Vitamin C has been found to significantly improve outcomes in these patients by reducing the severity of sepsis, decreasing organ dysfunction, and lowering mortality rates^[38]^. For instance, studies have demonstrated that high-dose intravenous vitamin C in septic patients can reduce the duration of vasopressor support and improve survival rates. These findings suggest that incorporating vitamin C into transfusion protocols for critically ill patients can lead to substantial clinical benefits and improved survival outcomes. Patients with chronic conditions such as sickle cell disease, thalassemia, and chronic anemia often require regular blood transfusions^[39]^. These patients are at risk of iron overload, oxidative damage, and immune complications from repeated transfusions. Vitamin C’s role in reducing oxidative stress and supporting immune function can be particularly beneficial in these contexts. By improving the quality of transfused blood and reducing the oxidative burden, vitamin C helps manage these chronic conditions more effectively. Patients experience fewer transfusion-related complications, better disease management, and enhanced quality of life.

One of the significant risks associated with blood transfusions is the increased susceptibility to infections, particularly in immunocompromised patients. Vitamin C’s immune-boosting properties help enhance the body’s defense mechanisms, reducing the risk of post-transfusion infections. Studies have shown that vitamin C supplementation can enhance the function of neutrophils, lymphocytes, and macrophages, thereby improving the overall immune response. This reduced infection rate is crucial for patients undergoing surgeries, cancer treatments, or those with weakened immune systems, leading to better clinical outcomes and fewer complications. Several clinical trials and studies have indicated that vitamin C can reduce mortality rates in patients receiving blood transfusions^[40,41]^. For instance, in patients with sepsis or severe infections, high-dose vitamin C has been linked to significant reductions in mortality. These outcomes are attributed to vitamin C’s ability to mitigate oxidative damage, support immune function, and enhance overall cellular health. Lower mortality rates are a direct indicator of the efficacy and safety of incorporating vitamin C into transfusion protocols, highlighting its potential to save lives and improve long-term outcomes. Beyond immediate clinical outcomes, vitamin C supplementation in transfusion medicine can lead to improved quality of life for patients. By reducing complications, enhancing recovery, and supporting overall health, patients experience fewer disruptions to their daily lives and greater well-being. This is particularly important for patients with chronic conditions who require frequent transfusions, as improved transfusion outcomes directly translate to better day-to-day functioning and reduced healthcare burdens. Improving clinical outcomes with vitamin C supplementation also has economic benefits. Reduced complications, shorter hospital stays, and fewer re-admissions lead to lower healthcare costs. Additionally, the enhanced preservation of blood products with vitamin C can lead to more efficient use of blood supplies, reducing wastage and improving resource utilization. These economic advantages are beneficial for healthcare systems, enabling better allocation of resources and improving overall healthcare delivery. The clinical benefits of vitamin C in transfusion medicine are supported by numerous clinical trials and studies. For example, a randomized controlled trial in critically ill patients demonstrated that high-dose intravenous vitamin C significantly reduced the incidence of organ failure and mortality. Another study in surgical patients showed that vitamin C supplementation reduced the risk of post-operative infections and accelerated recovery. These and other studies provide robust evidence for the clinical efficacy of vitamin C, underscoring its potential to enhance transfusion outcomes.

## Potential mechanisms of vitamin C in transfusion medicine

Vitamin C (ascorbic acid) has garnered attention for its potential benefits in transfusion medicine due to its multifaceted effects on erythrocyte preservation, immune modulation, and overall clinical outcomes. Understanding the potential mechanisms through which vitamin C exerts these effects is essential for optimizing its use in blood transfusion protocols. Here, we explore the various mechanisms by which vitamin C may influence transfusion medicine, focusing on its roles as an antioxidant, immune modulator, and cellular health enhancer.

## 1. Antioxidant protection

One of the most well-established mechanisms of vitamin C is its role as a potent antioxidant. Vitamin C neutralizes ROS and free radicals that accumulate during erythrocyte storage and blood transfusions^[42]^. Vitamin C donates electrons to ROS, converting them into less harmful molecules. This process prevents oxidative damage to erythrocytes, reducing hemolysis and preserving cell membrane integrity. Vitamin C helps regenerate other antioxidants such as vitamin E and glutathione. By recycling oxidized vitamin E and maintaining cellular redox balance, vitamin C enhances the overall antioxidant defense system of erythrocytes and other cells involved in transfusion processes.

## 2. Maintenance of erythrocyte membrane integrity

Vitamin C contributes to the structural and functional stability of erythrocytes, which is crucial for maintaining the quality of blood products during storage^[43]^. Vitamin C inhibits lipid peroxidation, a process where ROS damage lipid membranes, leading to cell membrane breakdown. By protecting erythrocyte membranes from oxidative damage, vitamin C helps maintain cell viability and functionality. Vitamin C maintains hemoglobin in its functional form, preventing oxidative damage to hemoglobin molecules. This stabilization ensures that erythrocytes retain their oxygen-carrying capacity throughout storage.

## 3. Support for collagen synthesis

Vitamin C plays a vital role in collagen synthesis, which indirectly supports transfusion medicine through its effects on vascular health and tissue repair^[44]^. Vitamin C is essential for the hydroxylation of proline and lysine residues in collagen. This biochemical process stabilizes the collagen triple helix, which is crucial for maintaining vascular integrity and facilitating wound healing. By supporting collagen synthesis, vitamin C helps maintain the structural integrity of blood vessels. Healthy blood vessels are essential for optimal blood flow and effective delivery of transfused erythrocytes to tissues.

## 4. Immune system modulation

Vitamin C modulates various aspects of the immune system, which can have significant implications for transfusion medicine^[45]^. Vitamin C enhances the function of neutrophils and macrophages, promoting their ability to engulf and destroy pathogens. This improved phagocytic activity helps reduce the risk of post-transfusion infections. Vitamin C influences the production of cytokines, balancing pro-inflammatory and anti-inflammatory responses. By modulating cytokine release, vitamin C helps manage the inflammatory reactions associated with transfusions, potentially reducing the incidence of complications such as TRALI and FNHTRs.

## 5. Reduction of transfusion-related immunomodulation (TRIM)

Vitamin C may mitigate TRIM, a condition where blood transfusions alter the recipient’s immune system, leading to complications. Vitamin C’s antioxidant properties help reduce oxidative stress, which is a contributing factor to alloimmunization. By decreasing oxidative damage, vitamin C lowers the risk of the recipient developing antibodies against donor antigens. Vitamin C counteracts the immune suppression that can result from blood transfusions, thereby maintaining the recipient’s ability to mount effective immune responses against pathogens^[46,47]^.

## 6. Promotion of cellular repair and regeneration

Vitamin C supports cellular repair mechanisms, which is beneficial for maintaining erythrocyte health and improving transfusion outcomes^[48]^. Vitamin C facilitates the repair of damaged cells and tissues through its role in collagen synthesis and antioxidant defense. This support is crucial for both the preservation of erythrocytes during storage and the health of transfused patients. By promoting cellular repair processes, vitamin C aids in the recovery of patients following transfusions, leading to better clinical outcomes and shorter recovery times.

## 7. Regulation of iron metabolism

Vitamin C affects iron metabolism, which can influence transfusion practices and patient health^[49]^. Vitamin C enhances the absorption of non-heme iron from the diet by reducing ferric iron (Fe3 +) to ferrous iron (Fe2 +). This improved iron absorption can be beneficial for patients with iron deficiency anemia undergoing frequent transfusions. While primarily known for enhancing iron absorption, vitamin C also helps in managing iron overload conditions by reducing oxidative damage caused by excess iron, which is relevant for patients with conditions like thalassemia who receive multiple transfusions.

## 8. Modulation of oxidative stress in transfusion medicine

Vitamin C helps manage oxidative stress in the context of blood transfusions, a crucial factor in preserving blood product quality and patient safety^[50]^. Vitamin C neutralizes ROS generated during blood storage and transfusion, thereby preventing oxidative damage to erythrocytes and other blood components. By reducing oxidative stress, vitamin C can extend the shelf life of stored blood products, ensuring that blood transfusions are safe and effective for longer periods.

## 9. Synergistic effects with other antioxidants

Vitamin C works synergistically with other antioxidants to provide comprehensive protection against oxidative damage^[51]^. Vitamin C regenerates oxidized vitamin E, which is another important antioxidant in blood products. This synergy enhances the overall antioxidant capacity of blood storage solutions and improves the quality of stored erythrocytes. Vitamin C supports the regeneration of glutathione, a key intracellular antioxidant. This cooperation helps maintain the redox balance within erythrocytes and other cells involved in transfusion medicine.

## 10. Potential for combination therapies

Vitamin C’s mechanisms of action open the door for combination therapies in transfusion medicine^[52]^. Vitamin C can be combined with other nutrients and therapies to enhance transfusion outcomes. For instance, combining vitamin C with vitamin E or selenium might offer broader protective effects against oxidative stress and improve blood storage conditions. Future research may explore innovative formulations of vitamin C combined with other therapeutic agents to optimize its benefits in transfusion medicine.

## Important findings from current research to demonstrate the magnitude of the vitamin C-induced improvements

Recent research has illuminated the significant impact of vitamin C in enhancing blood transfusion practices, particularly through its antioxidative properties that mitigate oxidative stress in stored blood components. A study published in 2015 investigated the role of vitamin C as an additive in plasma during blood storage. The findings indicated that vitamin C supplementation effectively reduced oxidative stress markers, thereby preserving the quality of stored plasma over time^[53]^. In 2022, a study examined the effects of high-dose ascorbic acid infusion in critically ill patients requiring transfusions. The results demonstrated that such supplementation significantly reduced oxidative stress and pro-inflammatory markers, while elevating anti-inflammatory markers. This suggests that vitamin C infusion can enhance patient outcomes by modulating inflammatory responses associated with transfusions.^[54]^ Another study explored the supplementation of stored red blood cells with uric and ascorbic acid. The research revealed that this combination protected stored red blood cells from oxidative damage, thereby improving their storability and post-transfusion recovery. This underscores the potential of vitamin C in maintaining the integrity of stored blood cells^[55]^. Collectively, these studies highlight the substantial benefits of vitamin C in blood transfusion contexts, including the preservation of blood component quality during storage and the enhancement of patient outcomes post-transfusion. The antioxidative and anti-inflammatory properties of vitamin C play a pivotal role in these improvements, offering promising avenues for optimizing transfusion practices.

## Advancements in analytical techniques and storage methodologies have significantly enhanced our understanding of vitamin C’s role in blood preservation

### Analytical techniques

High-performance liquid chromatography (HPLC): The development of simplified HPLC methods with ultraviolet detection has facilitated accurate measurement of plasma vitamin C levels. These methods are cost-effective, require fewer reagents, and offer greater sample stability, making vitamin C analysis more accessible in clinical settings^[56]^.

### Storage methodologies

Encapsulation techniques: Encapsulating vitamin C in chitosan-based nanoparticles has been shown to improve its stability during storage. This method prolongs the release of vitamin C under physiological conditions, ensuring sustained antioxidative effects during blood storage^[57]^.

Supercooled storage with antioxidants: Incorporating antioxidants like vitamin C into storage solutions has been explored to enhance the preservation of RBCs. Studies suggest that supplementing storage media with vitamin C can mitigate oxidative damage, thereby improving RBC storability and post-transfusion recovery^[17,58]^.

## Vitamin C’s effectiveness with other antioxidants used in transfusion medicine

In transfusion medicine, the effectiveness of antioxidants such as vitamin C, vitamin E, and glutathione has been extensively studied for their roles in mitigating oxidative stress during blood storage. While each offers unique benefits, vitamin C stands out in certain contexts due to its distinct properties and mechanisms of action^[56]^.

### Vitamin C (ascorbic acid)

Vitamin C is a water-soluble antioxidant with a high reactivity toward reactive oxygen species (ROS). Its ability to donate electrons neutralizes free radicals efficiently, making it highly effective in preventing lipid peroxidation and maintaining the integrity of RBC membranes during storage. Furthermore, vitamin C regenerates other antioxidants, such as glutathione and vitamin E, creating a synergistic antioxidative effect. Its versatility in both plasma and cellular compartments enhances its overall utility in blood preservation^[57]^.

### Vitamin E (tocopherol)

Vitamin E, a lipid-soluble antioxidant, is particularly effective in protecting the lipid bilayer of cell membranes from peroxidation. However, its hydrophobic nature limits its activity to lipid-rich environments, making it less effective in aqueous solutions such as plasma or intracellular fluids. Additionally, vitamin E relies on co-antioxidants like vitamin C for regeneration after neutralizing ROS, which may limit its standalone effectiveness^[17]^.

### Glutathione

Glutathione, a tripeptide composed of glutamine, cysteine, and glycine, functions primarily as an intracellular antioxidant. It protects cells by reducing hydrogen peroxide and lipid peroxides, relying on enzymatic pathways for its activity. While glutathione is crucial in mitigating oxidative stress, its activity is confined to cells with active glutathione peroxidase systems, and its stability during storage is a challenge^[58]^.

## Why vitamin C might be preferred:

1. Broad-spectrum activity: Vitamin C operates in both lipid and aqueous phases, offering comprehensive protection across plasma and cellular compartments.
2. Regeneration capability: It regenerates oxidized forms of other antioxidants, such as vitamin E and glutathione, amplifying their antioxidative effects.
3. Stability and accessibility: Vitamin C is more stable and easier to integrate into storage media compared to glutathione, which requires enzymatic activity to function effectively.
4. Cost-effectiveness: Vitamin C is more cost-efficient and widely available compared to vitamin E and specialized glutathione formulations.
5. Dual action in donors and recipients: In addition to preserving stored blood, vitamin C supplementation in recipients has shown benefits in reducing oxidative stress and improving immune response post-transfusion^[58]^.

## How vitamin C could interact with anti-inflammatory agents to further reduce inflammation and improve patient outcomes

Vitamin C’s synergistic potential with anti-inflammatory agents represents a promising avenue for enhancing therapeutic outcomes in transfusion medicine and broader clinical settings. Combining vitamin C’s antioxidative and immunomodulatory properties with targeted anti-inflammatory agents could significantly reduce inflammation, improving patient recovery and minimizing complications post-transfusion.

## Mechanisms of synergy

1. Neutralization of oxidative stress:Oxidative stress and inflammation are intricately linked, as excessive ROS generation exacerbates inflammatory pathways. Vitamin C directly scavenges ROS and regenerates other antioxidants like vitamin E and glutathione, mitigating oxidative stress at its source. When combined with anti-inflammatory agents, such as corticosteroids or nonsteroidal anti-inflammatory drugs (NSAIDs), vitamin C can help attenuate the upstream triggers of inflammation, leading to a more comprehensive reduction in inflammatory markers^[57]^.
2. Modulation of pro-inflammatory cytokines:Vitamin C has been shown to downregulate pro-inflammatory cytokines such as interleukin-6 (IL-6) and tumor necrosis factor-alpha (TNF-α). When used alongside agents like TNF inhibitors or interleukin antagonists, vitamin C can amplify the suppression of these cytokines, providing enhanced control over the inflammatory cascade^[17]^.
3. Enhanced vascular protection:Inflammatory conditions often impair endothelial function, increasing vascular permeability and exacerbating tissue damage. Vitamin C supports endothelial integrity by reducing oxidative damage to endothelial cells and preserving nitric oxide bioavailability. Coupled with anti-inflammatory drugs that target endothelial inflammation, this dual action helps restore vascular function and reduces the risk of complications such as organ dysfunction^[58]^.
4. Immune cell regulation:Vitamin C influences the activity of neutrophils, macrophages, and lymphocytes, optimizing their function during immune responses. Anti-inflammatory agents targeting these cells can work synergistically with vitamin C to modulate excessive immune activation without compromising overall immunity, ensuring a balanced response that promotes healing.

## Clinical applications

- Critically ill patients:In intensive care settings, where oxidative stress and inflammation are prevalent, combining high-dose vitamin C with corticosteroids has demonstrated significant reductions in markers of systemic inflammation. Studies report improved survival rates and faster recovery in patients receiving this combination therapy.
- Chronic inflammatory conditions:In patients with chronic conditions requiring repeated transfusions, such as sickle cell anemia or thalassemia, the addition of vitamin C to anti-inflammatory regimens may prevent cumulative inflammatory damage while enhancing the efficacy of the treatment.
- Post-transfusion inflammation:For patients experiencing transfusion-related inflammation, such as TRALI, a vitamin C and anti-inflammatory agent regimen could provide dual benefits by reducing oxidative lung damage and controlling the associated inflammatory response^[58]^.

## The effects of vitamin C in comparison to conventional blood storage practices

The integration of vitamin C into blood storage practices has emerged as a promising adjunct to conventional methods, addressing some limitations of traditional preservation strategies while enhancing the quality of stored blood products. Current storage protocols primarily aim to maintain the viability and functionality of RBCs, platelets, and plasma, yet oxidative damage and biochemical changes, collectively referred to as the “storage lesion,” can compromise the efficacy of transfused components. Vitamin C offers a novel solution to mitigate these challenges^[56]^.

## Conventional blood storage practices

Conventional blood storage relies on anticoagulant-preservative solutions like CPD (Citrate-Phosphate-Dextrose) and additive solutions (e.g., SAGM – Saline, Adenine, Glucose, Mannitol). These solutions extend the shelf life of blood by maintaining osmotic balance, preventing coagulation, and providing metabolic substrates to RBCs. Prolonged storage generates ROS that damage cell membranes and hemoglobin, reducing RBC deformability and oxygen-carrying capacity. Loss of 2,3-DPG (2,3-diphosphoglycerate) and ATP impairs RBC functionality and survivability post-transfusion. Oxidative stress accelerates the shedding of microvesicles from RBCs, which can trigger inflammatory responses in recipients^[57]^.

## The role of vitamin c in enhancing preservation

Vitamin C, with its potent antioxidant properties, directly addresses these limitations by reducing oxidative stress and preserving cellular integrity.

1. Reduction of oxidative damage

Vitamin C scavenges ROS, preventing oxidative damage to phospholipid membranes and hemoglobin. This reduces hemolysis rates during storage and maintains the structural integrity of RBCs. By stabilizing the membrane, vitamin C decreases microvesicle formation, reducing potential post-transfusion inflammatory complications.

2. Maintenance of metabolic function

Vitamin C preserves intracellular levels of ATP and 2,3-DPG by protecting metabolic enzymes from oxidative inactivation. This enhances the oxygen-releasing capacity of stored RBCs, ensuring better functionality upon transfusion.

3. Synergistic effects with additive solutions

Studies have shown that supplementing storage media with vitamin C can enhance the efficacy of existing preservative solutions like SAGM. It complements mannitol’s role in preventing osmotic stress by reducing oxidative stress synergistically.

4. Improved platelet and plasma quality

For platelets and plasma, vitamin C prevents the oxidation of clotting factors and platelet surface proteins, _ensuring_ better hemostatic function. This is particularly beneficial for platelet-rich plasma and cryopreserved plasma^[17]^.

## Comparative studies: vitamin C vs. conventional storage

Research comparing vitamin C-enhanced storage to conventional practices has demonstrated measurable improvements:

- Lower hemolysis rates: Studies have reported a significant reduction in hemolysis and free hemoglobin levels in vitamin C-treated stored blood.
- Enhanced post-transfusion survival: RBCs stored with vitamin C exhibit higher post-transfusion recovery rates, translating to improved efficacy in clinical settings.
- Reduction in inflammatory responses: Vitamin C supplementation reduces the inflammatory cytokine release associated with transfusion of oxidatively damaged blood^[58]^.

## Specific dosage recommendations for vitamin C when used in a transfusion setting or as part of parenteral nutrition

The use of vitamin C in transfusion settings or as part of parenteral nutrition requires careful consideration of its dosage to maximize therapeutic benefits while avoiding potential adverse effects. Current research and clinical guidelines provide a foundation for specific dosage recommendations, tailored to the clinical context.

## Vitamin C dosage in transfusion settings

In the context of blood preservation and transfusion, vitamin C acts as an antioxidant to reduce oxidative damage and improve the quality of stored blood components. Studies and experimental protocols suggest the following dosage ranges:

- Storage media supplementation
  - Concentrations of 0.1 to 1 mM (17.5 to 175 mg/L) of vitamin C have been shown to significantly reduce oxidative stress and hemolysis during blood storage.
  - Optimal doses should align with the volume of blood stored (e.g., approximately 50–200 mg per unit of packed RBCs).
- Recipient therapy during transfusion
  - Intravenous vitamin C administration of 1 to 3 grams daily in divided doses may be used to reduce systemic oxidative stress and inflammation, particularly in critically ill or septic patients receiving transfusions^[17]^.

## Vitamin C in parenteral nutrition

For patients receiving total parenteral nutrition (TPN), vitamin C is a critical component to prevent deficiencies and support immune function and tissue repair. Recommended doses include:

- Maintenance dose
  - For general use, 100–200 mg/day of vitamin C is typically included in TPN formulations to meet daily nutritional needs.
- Therapeutic dose for critically ill patients
  - Higher doses, such as 500–1500 mg/day, may be administered intravenously to address acute oxidative stress, inflammation, or critical illness.

## Upper limits and safety considerations

Health authorities have established tolerable upper intake levels (ULs) for vitamin C to prevent adverse effects:

- Oral intake
  - The UL for oral vitamin C intake in adults is set at 2000 mg/day by the U.S. National Institutes of Health (NIH). Exceeding this may result in gastrointestinal discomfort or kidney stone formation in susceptible individuals. • Parenteral administration
-
  - Intravenous doses of up to 10 grams/day have been safely administered in clinical trials, particularly in oncology and critical care settings. However, these high doses should be used under strict medical supervision, especially in patients with renal impairment or glucose-6-phosphate dehydrogenase (G6PD) deficiency, due to the risk of hemolysis^[58]^.

## Tailoring dosages to patient needs

- For patients with normal renal function and no contraindications, moderate doses of vitamin C are safe and effective in transfusion and TPN settings.
- In critically ill patients, higher therapeutic doses may be warranted but should be guided by clinical assessment and monitoring for potential side effects.

## Specific patient populations that may be more susceptible to adverse effects from vitamin C

While vitamin C is generally safe for most individuals, certain patient populations may be more susceptible to adverse effects due to underlying health conditions, genetic factors, or interactions with other treatments. In clinical settings such as transfusion medicine and parenteral nutrition, it is essential to recognize these at-risk groups to ensure that vitamin C supplementation is used safely.

## 1. Patients with renal impairment

Patients with compromised renal function, including those with chronic kidney disease (CKD) or acute kidney injury, are at increased risk for vitamin C toxicity. This is because the kidneys play a central role in the excretion of excess vitamin C. When renal function is impaired, the body may struggle to eliminate higher doses of vitamin C, leading to:

- Kidney stone formation: Excess vitamin C is metabolized into oxalate, which can form crystals and contribute to kidney stones, especially in patients with pre-existing kidney problems.
- Renal dysfunction exacerbation: High vitamin C doses can further strain the kidneys, potentially leading to a decline in renal function over time.

In these patients, it is crucial to monitor vitamin C intake closely, and lower doses (often under 500 mg/day) should be considered to prevent complications^[56]^.

## 2. Individuals with G6PD deficiency

Glucose-6-phosphate dehydrogenase (G6PD) deficiency is a hereditary condition that impairs red blood cells’ ability to handle oxidative stress. Vitamin C, although an antioxidant, can exacerbate oxidative damage in individuals with G6PD deficiency, leading to hemolytic anemia. This is particularly concerning during periods of acute illness or when higher doses of vitamin C are used. G6PD-deficient individuals are at risk of:

- Hemolysis: Oxidative stress, especially in response to high doses of vitamin C, can trigger the premature destruction of red blood cells in these patients, leading to severe anemia.
- Acute episodes: During episodes of hemolysis, these patients can develop jaundice, fatigue, and complications requiring urgent medical attention.

Therefore, patients with G6PD deficiency should generally avoid high doses of vitamin C, especially above 200 mg/day, unless prescribed under medical supervision^[57]^.

## 3. Individuals with a history of kidney stones

Beyond renal impairment, individuals with a personal or family history of calcium oxalate kidney stones may be at an increased risk when taking high doses of vitamin C. As vitamin C is metabolized into oxalate, excessive intake can contribute to the formation of kidney stones, leading to:

- Stone formation and pain: Elevated oxalate levels can precipitate as calcium oxalate stones, causing excruciating pain and potential blockages in the renal tract.
- Recurrent stone formation: Patients with a history of recurrent kidney stones should be monitored closely when receiving vitamin C, especially when given in high doses (above 1000 mg/day).

In such cases, vitamin C supplementation should be limited, and patients should be encouraged to increase hydration and monitor urinary pH levels^[17]^.

## 4. Infants and young children

Infants, particularly premature babies or those with underlying medical conditions, may be more sensitive to vitamin C supplementation. Though vitamin C is essential for growth and immune function, excessive intake can lead to:

- Metabolic disturbances: Infants’ kidneys are not fully matured, which could impair their ability to clear excess vitamin C.
- Gastrointestinal issues: High doses of vitamin C may cause gastrointestinal upset, including diarrhea, which can further exacerbate dehydration in young children.

The recommended daily intake for vitamin C in children is typically well below adult levels, and supplementation should be approached cautiously, particularly in those who are vulnerable or under medical care^[58]^.

## 5. Diabetic patients

Diabetic patients, particularly those with poorly controlled blood glucose levels, may be at risk of hyperglycemia when consuming high doses of vitamin C. While vitamin C is generally considered safe for diabetic patients, its potential effects on glucose metabolism should be monitored, particularly with higher intravenous doses. Excessive vitamin C may:

- Interfere with glucose testing: High levels of vitamin C can interfere with certain blood glucose tests, leading to inaccurate readings and potential mismanagement of insulin therapy.
- Alter insulin sensitivity: In some studies, high doses of vitamin C have been shown to affect insulin sensitivity, which may complicate blood glucose control.

Although not an absolute contraindication, vitamin C doses in diabetics should be carefully considered and adjusted to avoid complications^[59]^.

## 6. Individuals with hemochromatosis

Hemochromatosis is a genetic disorder where iron accumulates in the body. Since vitamin C enhances the absorption of iron from the gastrointestinal tract, patients with hemochromatosis are at risk of exacerbating iron overload. The combination of high vitamin C levels and excess iron could lead to:

- Tissue damage: Excess iron can accumulate in organs like the liver, heart, and pancreas, leading to tissue damage, cirrhosis, heart failure, and diabetes.
- Increased risk of organ damage: High doses of vitamin C may promote further iron absorption and increase the likelihood of complications associated with iron overload.

For patients with hemochromatosis, it is critical to monitor vitamin C intake and limit it to safer doses (typically less than 500 mg/day), particularly during active management of iron overload^[15]^.

## Safety and adverse effects of vitamin C in transfusion medicine

In the context of transfusion medicine, vitamin C may be administered as part of parenteral nutrition to patients, but it is not a standard component of blood transfusions^[53]^. However, it’s important to understand the safety and potential adverse effects of administering vitamin C in a medical setting. Vitamin C is generally recognized as safe when administered in appropriate doses. It is a water-soluble vitamin, and excess amounts are excreted in the urine. Vitamin C is necessary for the synthesis of collagen, absorption of iron, and maintenance of healthy skin, blood vessels, and connective tissues. Patients receiving blood transfusions or who are critically ill may require vitamin C as part of their nutritional support. Vitamin C acts as an antioxidant, helping to neutralize harmful free radicals in the body. This can be beneficial in certain medical conditions. Vitamin C is important for wound healing and tissue repair, which may be relevant in the context of surgery or trauma patients who require blood transfusions.

In rare cases, some individuals may experience allergic reactions to vitamin C, especially when administered intravenously^[54]^. These reactions can include rash, hives, itching, swelling, and difficulty breathing. This is more common when vitamin C is administered in high doses or in a concentrated form. High oral doses of vitamin C can cause gastrointestinal discomfort, including diarrhea, cramps, and nausea. When given intravenously, gastrointestinal side effects are less likely. Excessive intake of vitamin C, especially in the form of supplements, has been associated with an increased risk of kidney stones in some individuals. This is less of a concern when administered intravenously under medical supervision. Vitamin C can interact with certain medications, affecting their absorption or effectiveness. Patients should inform their healthcare providers of all medications and supplements they are taking. Vitamin C enhances the absorption of non-heme iron (found in plant-based foods) but can interfere with the absorption of certain medications and reduce the efficacy of iron chelation therapy in individuals with iron overload disorders.

## Potential limitations or concerns regarding the use of vitamin C

Despite the promising benefits of vitamin C in clinical settings, its use comes with several potential limitations and concerns that must be carefully considered. These challenges primarily revolve around issues of dosing, patient-specific factors, and the broader impact on health outcomes.

## 1. Risk of overdose and toxicity

One of the primary concerns with vitamin C supplementation is the risk of overdose, particularly when high doses are administered intravenously or in patients with compromised kidney function. Excessive vitamin C can overwhelm the body’s ability to excrete it, potentially leading to:

- Gastrointestinal disturbances: High doses of vitamin C are known to cause stomach irritation, diarrhea, and nausea. These effects can complicate patient management, especially in those who are critically ill or undergoing multiple therapies.
- Kidney stones: The metabolism of vitamin C into oxalate can contribute to the formation of kidney stones, especially in patients with pre-existing renal conditions or a history of stone formation. This is a significant concern in the use of vitamin C for patients with impaired renal function, necessitating careful dosing and monitoring^[60]^.

## 2. Interference with medical conditions and treatments

Vitamin C has the potential to interact with various medical conditions and treatments, which could affect its safety and efficacy:

- Hemochromatosis: In patients with hemochromatosis, vitamin C enhances iron absorption, which can exacerbate iron overload. This increases the risk of tissue damage and organ failure due to excessive iron accumulation, complicating the management of these patients.
- G6PD deficiency: Individuals with glucose-6-phosphate dehydrogenase (G6PD) deficiency are at risk of hemolytic anemia when exposed to high doses of vitamin C. This oxidative stress could trigger premature destruction of red blood cells, leading to severe anemia and other complications.
- Diabetic patients: High doses of vitamin C might interfere with blood glucose measurements, leading to inaccurate readings and potential mismanagement of insulin therapy. There is also some evidence to suggest that vitamin C may alter insulin sensitivity, which could complicate blood glucose control, particularly in poorly controlled diabetics^[61,62]^.

## 3. Variability in patient response

The response to vitamin C supplementation can vary greatly among individuals due to factors such as genetics, underlying health conditions, and concurrent medication use. For instance, while vitamin C might provide substantial benefits in reducing oxidative stress and improving blood preservation in healthy individuals, these effects may be diminished or exacerbated in patients with specific genetic or metabolic conditions. This variability can make it challenging to establish universally effective dosages and therapeutic protocols^[5]^.

## 4. Lack of standardized protocols

Another significant limitation is the lack of standardized dosing protocols and guidelines for the use of vitamin C in clinical settings. While research supports its benefits, especially in transfusion medicine and parenteral nutrition, there is insufficient consensus on the optimal dosage and timing of administration. This lack of uniformity can lead to inconsistencies in clinical practice and complicate efforts to ensure the safe and effective use of vitamin C.

## 5. Potential for false positive results in diagnostic tests

Vitamin C, particularly when administered in high doses, can interfere with certain diagnostic tests, leading to false results. For example:

- Blood glucose testing: Vitamin C can interfere with some glucose assays, leading to falsely elevated glucose levels. This may result in inaccurate readings that could misguide treatment decisions, particularly in patients with diabetes or those receiving critical care.
- Urine tests: High doses of vitamin C might also affect urine test results, potentially skewing the diagnosis of conditions like urinary tract infections or kidney function^[49]^.

## 6. Limited long-term safety data

While short-term use of vitamin C is generally regarded as safe, the long-term safety profile, especially at high doses, remains unclear. Studies investigating long-term supplementation, particularly in critically ill or chronically ill patients, are limited. There is a need for further research to evaluate potential long-term adverse effects, especially in terms of organ function and overall patient health.

## 7. Cost and accessibility

While vitamin C supplementation is relatively inexpensive, the formulation and delivery method – particularly intravenous administration – can increase treatment costs. In resource-limited settings or for patients with limited access to healthcare, the additional expense of vitamin C administration may be a barrier to its widespread use. Additionally, some specialized formulations required for blood preservation may not be readily available or feasible in certain clinical environments^[49]^.

## Implications of vitamin C for transfusion practice

The implications for transfusion practice of vitamin C are primarily related to its use in specific clinical situations where patients may benefit from vitamin C supplementation in conjunction with blood transfusions^[55]^. Critically ill patients in intensive care units (ICUs) or those who require massive blood transfusions, such as trauma patients, burn victims, or those with severe sepsis, may have altered vitamin C metabolism. These patients may have lower levels of vitamin C and may benefit from supplementation to support their immune function and overall health. Vitamin C can help mitigate oxidative stress and improve endothelial function. Patients who receive blood transfusions often require surgery or have traumatic injuries. Vitamin C is essential for collagen synthesis and wound healing. Administering vitamin C in conjunction with blood transfusions may aid in the recovery and wound healing process. Vitamin C acts as an antioxidant and can help reduce oxidative damage, particularly in patients who experience ischemia-reperfusion injury, as seen in some surgical procedures and organ transplantation. Administering vitamin C may help protect tissues and organs during these procedures.

Vitamin C is known to support the immune system. In some cases, blood transfusion recipients may have compromised immune systems due to underlying conditions. Supplementing with vitamin C can help support the immune response. Vitamin C enhances the absorption of non-heme iron from plant-based foods. In patients who are anemic or who require iron supplementation in conjunction with blood transfusions, vitamin C can be administered to improve iron absorption. Some studies have suggested that vitamin C supplementation may reduce the risk of transfusion-related complications, such as acute lung injury (TRALI), in certain patient populations. The use of vitamin C in transfusion medicine should be approached on a case-by-case basis. The dosage and administration should be determined by healthcare professionals based on the patient’s clinical condition and specific needs^[56,57]^.

Table 1 shows the summary of the key points of the review article on the vitamin C paradigm.

**Table 1:** The vitamin C paradigm: new frontiers in blood transfusion paradigm

Topic	Key points
Introduction	Vitamin C’s role in blood transfusion medicine, focusing on its antioxidant properties and potential benefits for improving blood quality and patient outcomes
Benefits of vitamin C in blood transfusion	Enhances the quality of stored blood, reducing oxidative stress, preserving red blood cell integrity, and improving immune function in patients receiving transfusions
Technological developments	Advances in analytical techniques (e.g., HPLC, LC-MS) and novel storage options (e.g., Vitamin C-enriched blood bags) that facilitate the study and application of Vitamin C in blood preservation
Comparison with other antioxidants	Compared to Vitamin E and glutathione, Vitamin C is more effective due to its broader range of antioxidant activities, lower cost, and ease of administration
Interaction with anti-inflammatory agents	Vitamin C’s synergy with anti-inflammatory agents such as corticosteroids can further reduce inflammation, enhance healing, and improve overall patient outcomes
Vitamin C vs. conventional blood storage	Compared to traditional storage practices, Vitamin C enhances red blood cell lifespan, reduces cellular damage, and may improve transfusion outcomes by preserving blood quality longer
Dosage recommendations	General recommendation for Vitamin C in transfusion settings or parenteral nutrition is 200–500 mg/day. Upper limits of 2000 mg/day (for healthy adults) are recognized by health authorities
Patient populations at risk	Patients with renal impairment, G6PD deficiency, a history of kidney stones, young children, diabetics, and individuals with hemochromatosis should be monitored for potential adverse effects
Limitations and concerns	Risks include overdose, kidney stone formation, interactions with medical conditions (e.g., hemochromatosis, G6PD deficiency), lack of standardized protocols, and limited long-term safety data
Conclusion	Vitamin C offers significant benefits in blood transfusion medicine, but careful monitoring and dose adjustment are essential, particularly for vulnerable patient populations. Further research is necessary to optimize its use

## Future perspectives on the use of vitamin C in blood transfusion medicine

As the understanding of vitamin C’s role in blood transfusion medicine continues to evolve, there are several exciting future directions for research and clinical application. While current evidence highlights the potential of vitamin C to enhance blood quality, reduce oxidative stress, and improve patient outcomes, several key areas require further exploration to maximize its clinical utility and safety^[17]^.

## 1. Optimizing dosage and delivery methods

One of the most pressing areas for future research is determining the optimal dosage of vitamin C for different clinical scenarios. While some studies suggest that low to moderate doses can provide antioxidant protection without significant adverse effects, high doses may offer additional benefits. However, there is no consensus on the ideal range, particularly for patients with underlying health conditions or those undergoing critical care. Future studies should focus on refining dosing protocols based on patient-specific factors, such as renal function, age, and pre-existing conditions, to minimize risks while maximizing benefits. Additionally, the development of more effective and patient-friendly delivery methods, such as oral supplementation or novel intravenous formulations, could increase accessibility and reduce the burden of hospital-based treatments. This could be particularly valuable for patients requiring long-term vitamin C supplementation or those with limited access to healthcare resources^[63]^.

## 2. Addressing patient-specific variability

Another important consideration is the variability in how patients respond to vitamin C supplementation. Factors such as genetics, existing comorbidities, and concurrent therapies can influence the effectiveness and safety of vitamin C in transfusion medicine. Future studies should investigate how genetic variations or metabolic disorders (e.g., G6PD deficiency, hemochromatosis) interact with vitamin C supplementation, and how to tailor therapies for these patient populations. Personalized treatment approaches could help optimize the therapeutic benefits while reducing the risk of adverse effects^[64]^.

## 3. Long-term safety and efficacy

While short-term use of vitamin C has demonstrated promising results, there is limited long-term data on its safety, particularly at higher doses. Understanding the long-term impact of vitamin C on organ function, particularly in critically ill patients or those undergoing repeated transfusions, is essential. Future research should focus on evaluating the cumulative effects of vitamin C supplementation over extended periods, with an emphasis on kidney function, glucose metabolism, and overall patient health. These studies could provide more definitive evidence to support the routine use of vitamin C in blood transfusion settings^[65]^.

## 4. Synergistic effects with other therapies

The potential synergy between vitamin C and other therapeutic agents, such as anti-inflammatory drugs, iron chelators, or antimicrobial treatments, offers a promising avenue for future exploration. For instance, vitamin C’s ability to reduce inflammation when combined with corticosteroids or other immunomodulatory therapies could further improve clinical outcomes for patients undergoing transfusions. Research exploring the combined effects of vitamin C and other antioxidants or drugs could lead to more comprehensive treatment strategies, particularly for patients with complex or multi-faceted health issues^[66]^.

## 5. Expanding use beyond blood transfusion

While vitamin C has primarily been studied in the context of blood storage and transfusion, its potential applications could extend to other areas of clinical medicine. For example, its role in improving immune function, enhancing wound healing, and reducing infection rates in hospitalized patients could broaden its therapeutic use. As research on vitamin C’s systemic effects continues to grow, clinicians may begin to incorporate it more frequently into broader treatment regimens for critically ill patients, improving overall outcomes in a variety of settings^[64]^.

## 6. Standardizing protocols and clinical guidelines

A significant barrier to the widespread use of vitamin C in blood transfusion medicine is the lack of standardized clinical protocols and guidelines. While promising, the variability in research findings and the absence of uniform treatment regimens have slowed the adoption of vitamin C in routine practice. Future research should aim to establish clear, evidence-based guidelines for its use, addressing dosage, delivery methods, monitoring protocols, and patient selection criteria. These guidelines would facilitate its incorporation into clinical practice, ensuring consistent and safe application^[65]^.

## 7. Cost-effectiveness and accessibility

Although vitamin C is relatively inexpensive, the cost of novel formulations and intravenous administration may limit its widespread use, particularly in resource-limited settings. Future studies should investigate the cost-effectiveness of vitamin C supplementation in transfusion medicine, considering both direct treatment costs and potential long-term savings from improved patient outcomes. Additionally, exploring more accessible delivery methods, such as oral supplementation or affordable vitamin C-enriched blood storage options, could increase its availability in low-resource healthcare settings^[66]^.

## 8. Integrating vitamin C into blood bank practices

As more research supports the benefits of vitamin C in preserving blood quality, future advancements may see its integration into routine blood bank practices. This could include incorporating vitamin C into blood storage solutions, developing optimized blood bags, or even using vitamin C as part of the processing protocol for blood donations. Such innovations could lead to longer shelf life for donated blood and improve the safety and efficacy of transfusions, particularly in patients who require frequent or long-term transfusions^[49]^.

## Recommendations for the use of vitamin C in blood transfusion medicine

Given the promising evidence surrounding the benefits of vitamin C in blood transfusion medicine, several key recommendations emerge for its optimal use, future research, and integration into clinical practice. These recommendations aim to address both current challenges and opportunities for maximizing vitamin C’s effectiveness while ensuring patient safety and improving overall outcomes.

## 1. Standardization of dosage and administration protocols

One of the most critical steps in optimizing vitamin C’s use in blood transfusion medicine is the development of standardized dosing and administration protocols. While current research suggests that vitamin C can offer antioxidant protection, the ideal dosage remains unclear, particularly for various patient populations, including those with renal impairments or underlying conditions like diabetes. It is recommended that clinical guidelines be developed to define safe and effective dosage ranges for different clinical contexts. For instance, routine supplementation in transfusion settings should consider a range of 200–500 mg per day, while critically ill patients or those with complex medical conditions might require tailored doses based on their individual needs. More robust clinical trials are necessary to provide clear guidance on the most effective and safest doses, both for blood preservation and patient supplementation during and after transfusion.

## 2. Patient-specific tailoring of vitamin C therapy

As highlighted by research, the response to vitamin C supplementation can vary significantly across different patient populations. Those with conditions such as renal dysfunction, G6PD deficiency, and hemochromatosis may be at higher risk for adverse effects from vitamin C. Therefore, it is crucial that clinicians tailor vitamin C therapy based on patient-specific factors. For patients with renal impairment, for example, dosing should be lower, and blood levels should be monitored closely to avoid complications like kidney stones. Similarly, patients with iron overload conditions (e.g., hemochromatosis) should be carefully assessed, as high doses of vitamin C can increase iron absorption and exacerbate the condition. Personalized treatment protocols that account for such conditions will help mitigate risks while ensuring the therapeutic benefits of vitamin C.

## 3. Further research on long-term safety and efficacy

While short-term studies have demonstrated vitamin C’s ability to improve blood quality and reduce oxidative stress, the long-term safety and efficacy of vitamin C, particularly at higher doses, remain underexplored. It is essential that future studies investigate the long-term effects of vitamin C supplementation, especially in patients undergoing repeated transfusions or critical care. This research should focus on assessing the cumulative impact of vitamin C on kidney function, glucose metabolism, and other systemic effects over extended periods. Additionally, large-scale longitudinal studies should explore the potential benefits of vitamin C in reducing transfusion-related complications, such as infections or immune reactions, to further solidify its role in clinical transfusion medicine.

## 4. Integration of vitamin C into blood storage practices

The promising effects of vitamin C on preserving red blood cell integrity and reducing oxidative damage open up the possibility of incorporating it into blood storage practices. Blood banks should consider investigating vitamin C-enriched blood bags or storage solutions to enhance the quality and shelf life of stored blood. This could be particularly beneficial for patients who require frequent or long-term transfusions, such as those with chronic anemia or sickle cell disease. Collaborative efforts between blood banks, research institutions, and pharmaceutical companies should be made to develop cost-effective and scalable methods for incorporating vitamin C into blood preservation. By integrating vitamin C into blood storage solutions, it is possible to enhance blood quality and reduce the incidence of transfusion-related complications.

## 5. Synergistic use with other therapeutic agents

Another promising avenue for future research is the exploration of synergistic effects between vitamin C and other therapeutic agents used in transfusion medicine, such as anti-inflammatory drugs, iron chelators, and antimicrobial treatments. Combining vitamin C with corticosteroids or other anti-inflammatory drugs could reduce inflammation more effectively and improve clinical outcomes in patients undergoing transfusions. Similarly, investigating the role of vitamin C alongside other antioxidants, like vitamin E or glutathione, could provide insight into their combined effects on preserving blood cells and improving immune function in transfusion recipients. These combination therapies may hold significant promise in enhancing patient outcomes, especially in high-risk or critically ill populations.

## 6. Increased focus on accessibility and cost-effectiveness

One of the main barriers to the widespread adoption of vitamin C in transfusion medicine, particularly in resource-limited settings, is its cost and accessibility. Although vitamin C itself is relatively inexpensive, novel formulations or intravenous delivery methods may present financial challenges. It is recommended that efforts be made to reduce the cost of vitamin C-based therapies and explore more affordable delivery options, such as oral supplementation or vitamin C-enriched blood storage options. Furthermore, large-scale studies on the cost-effectiveness of vitamin C in transfusion medicine could help demonstrate its potential economic benefits, such as reducing transfusion-related complications or improving recovery times, which could ultimately lower healthcare costs.

## 7. Collaboration and multidisciplinary research

Given the potential of vitamin C to revolutionize transfusion medicine, it is essential for researchers, clinicians, and healthcare professionals to collaborate in a multidisciplinary approach. This includes the integration of knowledge from fields such as hematology, immunology, pharmacology, and nutrition to better understand how vitamin C can be most effectively used in transfusion medicine. Collaborations with pharmaceutical companies to develop novel vitamin C formulations and partnerships with blood banks to explore the benefits of vitamin C-enriched storage solutions will be key to advancing this area of research. By working together, the medical community can ensure that vitamin C’s full therapeutic potential is realized, benefiting patients globally.

## 8. Education and training for healthcare providers

Finally, educating healthcare providers about the benefits and safe use of vitamin C in transfusion medicine is essential for successful integration into clinical practice. Clinicians should be informed about the latest research, safe dosage recommendations, and potential patient-specific risks associated with vitamin C supplementation. Continuous education programs, clinical workshops, and access to updated clinical guidelines will help ensure that vitamin C is used effectively and safely, ultimately improving patient care in blood transfusion settings.

## Conclusion

Vitamin C plays a crucial role in various clinical settings, including blood transfusion medicine and parenteral nutrition, due to its antioxidant properties and potential to improve blood quality and patient outcomes. However, certain patient populations – such as those with renal impairment, G6PD deficiency, a history of kidney stones, young children, diabetics, and individuals with hemochromatosis – are more susceptible to adverse effects from excessive vitamin C intake. Future research is needed to further clarify the optimal dosage and administration protocols for these high-risk groups. Additionally, prospective studies should explore the long-term impact of vitamin C supplementation in these populations, assessing not only its therapeutic benefits but also its safety in various clinical contexts. Enhanced monitoring strategies and individualized care plans will be crucial to maximizing the benefits of vitamin C while minimizing the potential risks, particularly in vulnerable patient populations. This will help to refine the guidelines for its use in transfusion settings and other therapeutic areas.

## Data Availability

Not applicable.
